# Seedling maize counting method in complex backgrounds based on YOLOV5 and Kalman filter tracking algorithm

**DOI:** 10.3389/fpls.2022.1030962

**Published:** 2022-11-07

**Authors:** Yang Li, Zhiyuan Bao, Jiangtao Qi

**Affiliations:** ^1^ Key Laboratory of Bionic Engineering, Ministry of Education, Jilin University, Changchun, China; ^2^ College of Biological and Agricultural Engineering, Jilin University, Changchun, China; ^3^ Key Laboratory of Tea Quality and Safety Control, Ministry of Agriculture and Rural Affairs, Tea Research Institute, Chinese Academy of Agricultural Sciences, Hangzhou, China

**Keywords:** object detection, YOLOv5, video tracking, maize plants, counting prediction

## Abstract

Maize population density is one of the most essential factors in agricultural production systems and has a significant impact on maize yield and quality. Therefore, it is essential to estimate maize population density timely and accurately. In order to address the problems of the low efficiency of the manual counting method and the stability problem of traditional image processing methods in the field complex background environment, a deep-learning-based method for counting maize plants was proposed. Image datasets of the maize field were collected by a low-altitude UAV with a camera onboard firstly. Then a real-time detection model of maize plants was trained based on the object detection model YOLOV5. Finally, the tracking and counting method of maize plants was realized through Hungarian matching and Kalman filtering algorithms. The detection model developed in this study had an average precision mAP@0.5 of 90.66% on the test dataset, demonstrating the effectiveness of the SE-YOLOV5m model for maize plant detection. Application of the model to maize plant count trials showed that maize plant count results from test videos collected at multiple locations were highly correlated with manual count results (*R^2^* = 0.92), illustrating the accuracy and validity of the counting method. Therefore, the maize plant identification and counting method proposed in this study can better achieve the detection and counting of maize plants in complex backgrounds and provides a research basis and theoretical basis for the rapid acquisition of maize plant population density.

## Introduction

Crop planting density counts the number of plants per unit area, which has a great impact on the yield and quality of crops and is one of the important factors of agricultural production systems (Zhi et al., 2016; Zhai et al., 2018; Adams et al., 2019; Chapepa et al., 2020; Ndou et al., 2021). The research on maize planting density plays an important role in early breeding decisions to improve yield (Zhai et al., 2018). Therefore, it is essential to estimate the population density of maize accurately and timely.

To estimate plant population densities, the traditional field assessments method counts the number of plants in a randomly selected partition manually of a field and uses the average of multiple partitions to express plant population density. This method is time-consuming, labor-intensive, and inaccurate. To solve this problem, some studies have used color RGB images to count crops in the field (Lv et al., 2019; Zhao et al., 2021; Qi et al., 2022). These studies are based on traditional image processing algorithms that primarily use color information to segment crop areas for crop counting. These methods have high counting accuracy (approximately 90%) under certain conditions but have the following shortcomings. Firstly, the color information is easily affected by the surrounding light intensity and crop status. For example, plants looked darker on cloudy days than on sunny days and may have different colors at different stages of growth. Secondly, some counting methods are closely related to location and time. Typically, these methods require the necessary calibration by manually counting plants in a small portion of the field to build a regression model between pixel counts and actual plant counts. Then the regression model was applied to the rest of the images to achieve automatic processing. Therefore, a regression model established at one site (or growth stage) usually cannot be applied directly to another site (or growth stage), and the model needs to be re-validated or calibrated at a new site (or growth stage).

In recent years, many crop detection and counting methods based on traditional image processing (Zhao et al., 2021), machine learning (Lv et al., 2019), and deep learning technology (Qi et al., 2022) have been studied. For the three types of methods mentioned above, traditional image processing methods are easily disturbed by factors such as illumination, noise, and weed background. The shallow features such as color, shape, and texture extracted by machine learning methods have limited expression ability, and lack universality and adaptability. Deep convolutional neural networks (CNN) have shown powerful performance in object detection for agricultural images in recent years (Zhao et al., 2019). Many algorithms based on deep learning models have been successfully applied to the detection of a variety of crops. For example, researchers have explored the use of models such as YOLO and Faster-RCNN for the detection of fruits (Koirala et al., 2019; Häni et al., 2020), trees (Zhou et al., 2021), and crops (Hu et al., 2013; Jin et al., 2019). These studies reported promising detection accuracy and thus per-image counting accuracy.

For the counting methods based on image sequences, how to prevent the repeated counting of the same object in a continuous image sequence is a key problem. Methods to address this problem can be divided into three main categories. The first class of methods uses 3D reconstruction techniques to reconstruct space point cloud information from 2D images, then detection and counting were made in the 3D space (Häni et al., 2020; Gené-Mola et al., 2020). Since a plant is unique in the 3D space, a plant that is repeatedly counted in 2D images will be highly overlapped in the 3D space. Therefore, repeated counting of a plant can be avoided in the 3D space. The second class of methods uses the position and pose information of the imaging device to estimate the geometric correspondence between the same target in two consecutive images (Stein et al., 2016). Using this method, objects detected in two images captured at different locations can be associated, then the objects could be tracked and counted. The third type of method is the tracking method based on the object detection results. The key to this method is to establish the associations between detection results and the trackers (Gao et al., 2022; Lin et al., 2022). The mentioned three types of methods can achieve high counting accuracy under certain conditions, but they have certain shortcomings and problems. The method based on 3D reconstruction technology has a high computational cost and the 3D reconstruction results are easily affected by the external environment. The computational cost of the second method is lower than that of the method based on 3D reconstruction techniques, but the applied sensors (e.g., RTK GPS) made the cost of systems becomes very high. The detection-based tracking counting method has a low cost, but the robustness of this strategy is still insufficient to a certain extent. Since the IoU threshold is obtained from a small portion of the image sequence data, the threshold may fail when the test image sequence is obtained in a different environment (Jiang et al., 2019). Recently, other new tracking strategies can handle this problem. For example, the research of tracking algorithms based on correlation filtering has made promising progress recently, especially in the Kalman filtering method (Wang et al., 2019; Zhang et al., 2022).

The target detection model YOLOv5 has fast detection speed, and many target tracking algorithm has been applied to the tracking and quantity statistics of vehicles and pedestrians recently. Research shows that YOLOv5 and detection-based tracking algorithm could quickly and accurately count objects in videos. At the same time, UAVs have shown great potential as remote sensing platforms for crop growth monitoring in recent years (Wang et al., 2019). So it is necessary to explore the research on the detection and counting of maize plants by combing of CNN and drones. In this study, the image datasets were collected by a low-altitude UAV first. Then the maize plants detection method based on the SE-YOLOV5m model was trained. And the trained SE-YOLOV5m model and Kalman filter algorithm were combined to track and count maize plants in individual videos. Finally, the counting method was tested and evaluated on test videos.

## Materials and methods

### Image acquisition and processing methods

**Figure 1:**
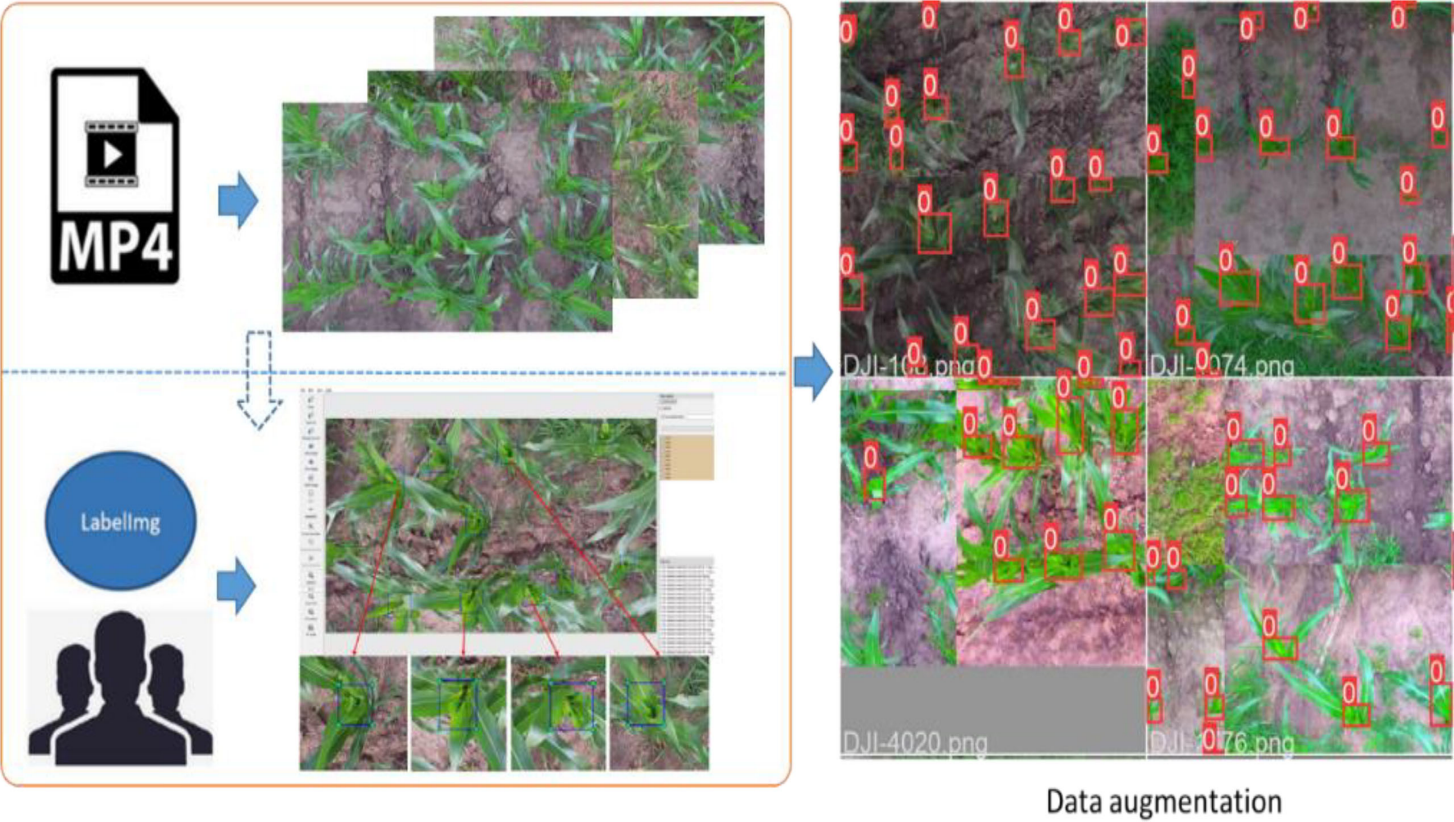
Data processing flow and data enhancement examples.

The DJI Phantom 4 was used for taking pictures of corn canopy. The Phantom 4 featured a fully stabilized 3-Axis gimbal system with a 4k 12-megapixel camera and up to 27 minutes of flight time. The collection site was Nong'an County, Changchun (125.153436 N, 44.166099 E). According to the identification system, maize development can be divided into vegetative (V) and reproductive (R) stages. The V stages are designated numerically as V(n), where (n) represents the number of leaves with visible collars. We collected videos for plants from stages V4 to V6, which are the vegetative growth stages of maize plants (Zea mays L., Jingke 968) when the fourth, fifth, and sixth leaf collars are visible. The images and videos containing the maize plants were taken in different weather conditions (cloudy and sunny) with the UAV flying at a height of approximately 4 meters. The width and height of the images were 3840 and 2160 pixels, respectively. The collected videos are divided into a detection dataset and a counting dataset according to the ratio of 6:4. Images were extracted every 10 frames from every video in the detection set. They were used to train and validate the detection model together with the collected images. And videos in the counting dataset were used to validate the performance of the final counting algorithm. The training samples were manually labeled using Labelimg software (Tzutalin, 2015). Since the size of the original images was 3840 and 2160 pixels, which were too large for labeling and training. So the original images were first cropped to 960 and 540 pixels, respectively. The maize plants between the V4 and V6 stages look like small bell mouths when viewed from the top. It is obviously different from the rest of the leaves in color, brightness, and shape, so this feature is mainly used as the labeling standard. Some labeled images are shown in **Figure 1**. After labeling, a total of 2200 images were obtained, which contains 22235 maize plants. The images in the detection dataset were split into the a training set, a validation set, and a test set in the ratio of 8:1:1. In order to prevent overfitting and improve the generalization ability of the model, several date augmentations methods were applied. Such as image perturbation, changing brightness, changing contrast, changing saturation, changing hue, adding noise, random scaling, random crop, flipping, rotating, random erasing, and so on. In addition, Mosaic (Glenn, 2022) was also used. The data processing flow and data enhancement examples are shown in **Figure 1**.

### Maize plants detection model

For the maize plant quantity statistics method proposed in this study, the first thing to study is the design of maize plant detection model. The model of YOLOv5 (Glenn, 2022) series is able to substantially improve the detection speed while maintaining the detection accuracy of existing models, and is one of the optimal choices for target detection. So the model of YOLOv5 series was used to build the maize plants detection model. The YOLOv5 model is an upgraded version based on YOLOv3 (Redmon and Farhadi, 2018). Four object detection models of different depths and widths can be trained by using the official code. The YOLOv5s has the smallest depth and width in the YOLOv5 series. The other three networks are deepened and broadened on the basis of it. The YOLOv5 directly uses a single neural network to predict and classify input images to achieve end-to-end object detection. And it proposes cross-scale prediction, which enables the network to detect objects at three different scale features and adapt to multiple object detection tasks of different sizes. The backbone and the neck of the model use CSPDarknet53 (Wang et al., 2020) and the PAN (Liu et al., 2018) structure, respectively. Two different CSP modules are used in different parts of the model. Specifically, the C3_x module is applied to the backbone, the other C3_F_x module is used in the later structure. Comparing the speed and accuracy of the four different YOLOv5 models in **Table 1**, it can be seen that the mAP of YOLOv5m is 2.9% higher than that of YOLOv5s, and 0.8% and 1.6% lower than that of the YOLOv5l model and the YOLOv5x model, respectively. On the other hand, the model size of YOLOv5m is 26.7 MB larger than that of YOLOv5s, but it is 1/2 and 1/4 of that of YOLOv5l and YOLOv5x, respectively. Therefore, after balancing the detection accuracy and the model size of the network, the YOLOv5m model was used as the base for research.

**Table 1:** Comparison of model prediction results.

Models	mAP (%)	Average detection speed (ms)	Model size (MB)
YOLOV5s	87.65	18.2	14.1
YOLOV5m	90.24	20.3	40.8
YOLOV5l	91.02	22.4	89.2
YOLOV5x	92.15	25.6	166

**Figure 2:**
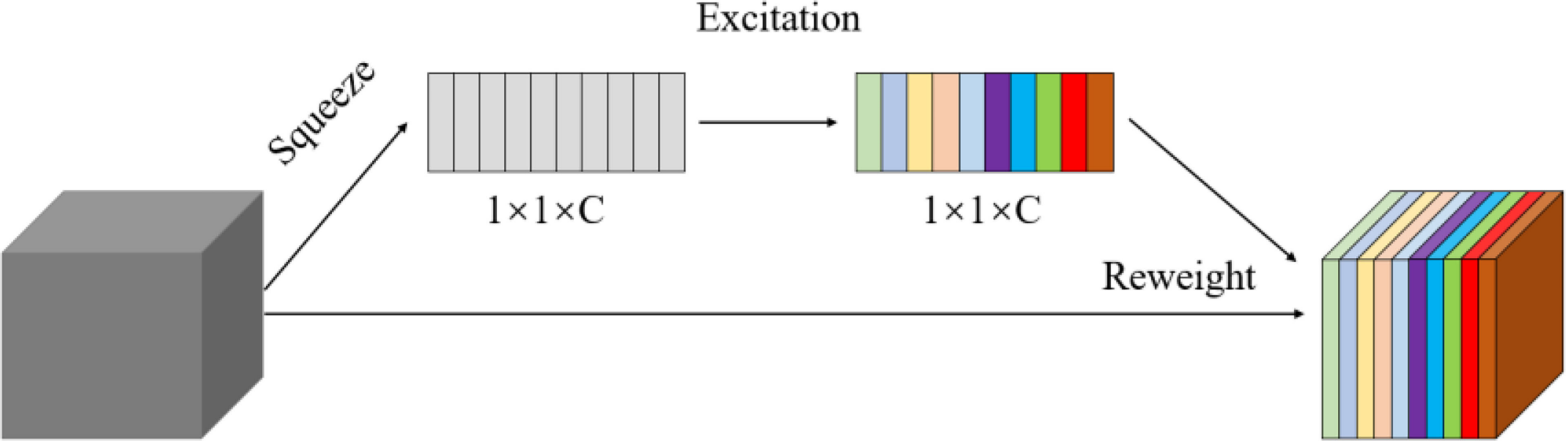
The structure of Squeeze and excitation (SE).

**Figure 3:**
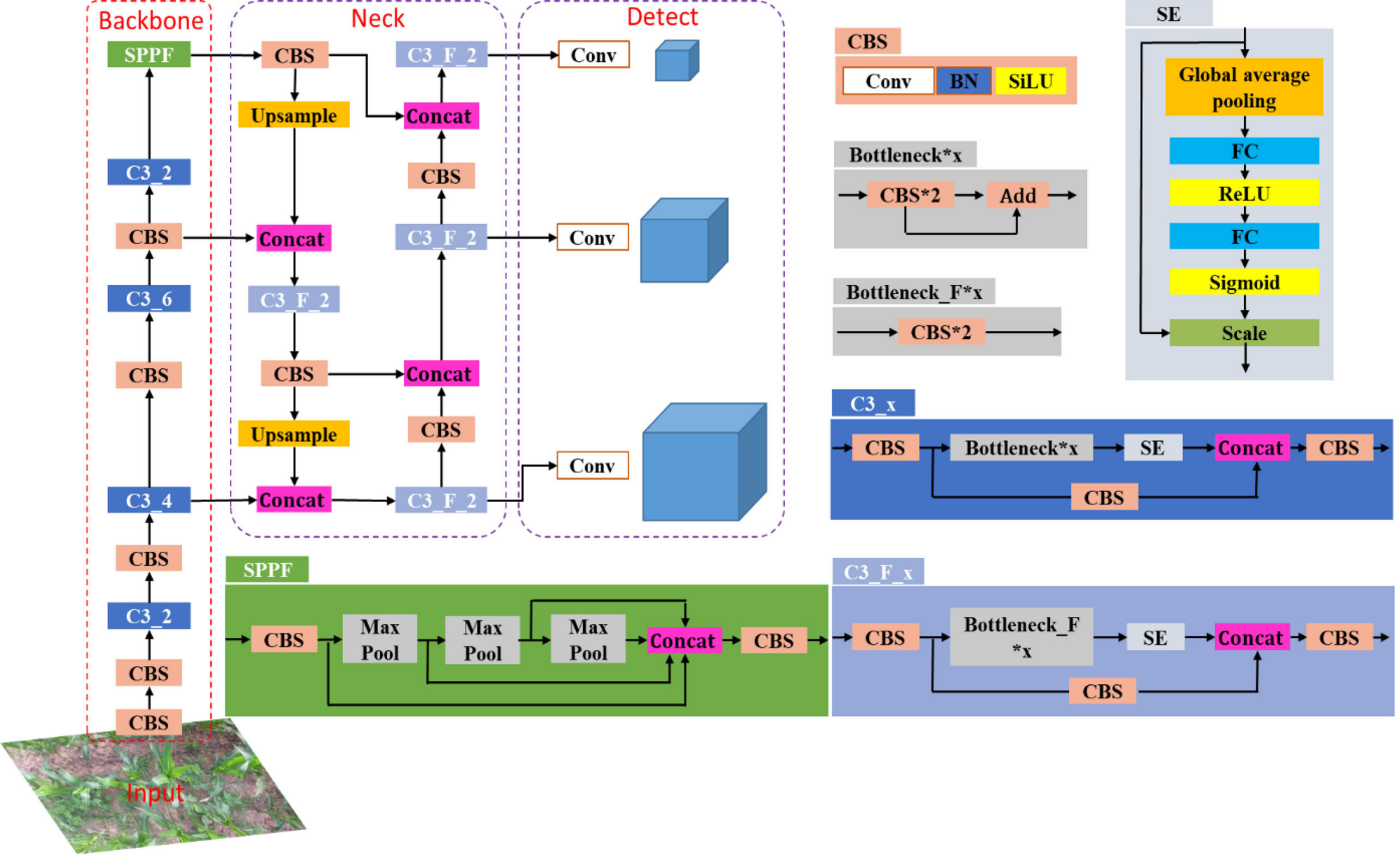
The structure of improved SE-YOLOV5m model. CBS contains a Conv, a BN and a SiLU (sigmoid liner relu) activation function, where Conv is 2D Convolutional layer, BN indicates batch normalization. C3_x indicates the use of a CBS structure with X residual modules (ResUnit), e.g. in the first C3_x, one residual components are used, hence C3_1. C3_F_X has the same meaning as C3_X.

Related research shows that visual attention mechanism can improve the accuracy of deep learning models (Yang et al., 2020). To improve the efficiency and accuracy of detecting maize plants, the Squeeze and Excitation Networks (SENet) (Hu et al., 2018) was introduced in the CNN. The SENet could obtain the weight of each channel of the features and then uses the weight to filter the key features, which could improve the representation capability of CNN. As shown in **Figure 2**, the SE module mainly contains squeeze and excitation operations (Hu et al., 2018). It performs a squeeze operation firstly, then performs an excitation on the global features to obtain the weights of different channels and the relationship between the channels. As shown in **Figure 3**, the structure of improved SE-YOLOv5m was proposed in this study. As shown in the figure, the SE module is embedded in the C3_x module and C3_F_x module individually. The purpose of the SE module is to enhance the feature extraction ability of the model by emphasizing the key feature of maize plants and suppressing background features to improve the detection accuracy in multiple scenarios.

### Counting model based on YOLOV5

Firstly, the YOLOV5 model was used to detect maize plants in continuous static images. Then a tracker based on Kalman filter (Kalman, 1960) was used to track the maize plants to avoid repeated counting of them in continuous image sequences. Based on the trackers, each maize plant would be given a unique tracking number, so that every maize plant would only be counted only once. The tracking counting model contains three steps: maize plants state estimation, association and matching of maize plants between frames, and trackers update.

#### Maize plants state estimation

To track each maize plant detected by the detection model, the following state variable was used to represent the status of the maize plants:


(1) $$\text{t}=(\text{u},\text{v},\text{s},\text{r},\dot{\text{u}},\dot{\text{v}},\dot{\text{s}})$$

where u, v, s, r are the horizontal and vertical coordinates of the center point of the plant bounding box in image coordinates (in pixels), the area of the bounding box (in pixels), and the aspect ratio, respectively. $\dot{u},\dot{v},\dot{s}$ are their corresponding first derivatives with respect to time in image coordinates.

The plant tracking problem is a discrete-time series problem and consists of the following two main steps: the first is prediction process. Through the Kalman filter dynamic model, the state variables of the maize plant in the current frame would be used to predict the state variables in the next frame. The second step is the update process. The observed variables (detected bounding box) of the maize plant in the next frame would be used to update the state variables predicted in the prediction process (Jiang et al., 2019). Since the camera has a high frame rate, the position change of the target between video sequences is very small. So the motion of the camera can be regarded as a uniform motion. Therefore, it is assumed that the visual detection and tracking system is linearly correlated with the time change. A standard Kalman filter with constant velocity motion and linear observation model was used, which takes a 4-dimensional state (u, v, s, r) as the direct observation model of the maize plant.

The state parameters u, v, s, r of the tracker are initialized according to the detection results in the first frame, and $\dot{u},\dot{v},\dot{s}$ are set to 0. After the first frame ( *i* ≥2 ), the state variables (t) and the state covariance matrix (***P***) of the trackers in the *i*th image are estimated using the data of the trackers in the (*i-1*)th image in the prediction process. The following formulas were used in the prediction process (Jiang et al., 2019):


(2) $${\hat{t}}_{k}^{i|i-1}=F{\hat{t}}_{k}^{i-1|i-1},F=\left[\begin{array}{ccccccc} 1 & 0 & 0 & 0 & 1 & 0 & 0\\ 0 & 1 & 0 & 0 & 0 & 1 & 0\\ 0 & 0 & 1 & 0 & 0 & 0 & 1\\ 0 & 0 & 0 & 1 & 0 & 0 & 0\\ 0 & 0 & 0 & 0 & 1 & 0 & 0\\ 0 & 0 & 0 & 0 & 0 & 1 & 0\\ 0 & 0 & 0 & 0 & 0 & 0 & 1 \end{array}\right]$$

(3) $$P_{i|i-1}=FP_{i-1|i-1}F^{T}+Q,Q=\left[\begin{array}{ccccccc} 1 & 0 & 0 & 0 & 1 & 0 & 0\\ 0 & 1 & 0 & 0 & 0 & 1 & 0\\ 0 & 0 & 1 & 0 & 0 & 0 & 1\\ 0 & 0 & 0 & 1 & 0 & 0 & 0\\ 0 & 0 & 0 & 0 & 10^{-2} & 0 & 0\\ 0 & 0 & 0 & 0 & 0 & 10^{-2} & 0\\ 0 & 0 & 0 & 0 & 0 & 0 & 10^{-4} \end{array}\right]$$

Where ${\hat{t}}_{k}^{i|i-1}$ is the *a priori* state estimate for the *k*th plant tracker in the *i*th frame, ${\hat{t}}_{k}^{i-1|i-1}$ is the a posteriori state estimate for the *k*th plant tracker in the (*i*-*1*)th frame, ***F*** is the state transfer matrix, *P*_*i*|*i*−1_ is the *a priori* state covariance matrix for the *i*th frame, *P*_*i*−1|*i*−1_ is the a posteriori state covariance matrix for the (*i*-*1*)th frame, and *Q* is the random process noise matrix.

The following formula is used to calculate the posterior state covariance matrix of the *i*th frame image and the posterior state of the tracker.


(4) $$S_{i}=HP_{i|i-1}H^{T}+R,H=\left[\begin{array}{ccccccc} 1 & 0 & 0 & 0 & 0 & 0 & 0\\ 0 & 1 & 0 & 0 & 0 & 0 & 0\\ 0 & 0 & 1 & 0 & 0 & 0 & 0\\ 0 & 0 & 0 & 1 & 0 & 0 & 0 \end{array}\right],R=\left[\begin{array}{cccc} 1 & 0 & 0 & 0\\ 0 & 1 & 0 & 0\\ 0 & 0 & 10 & 0\\ 0 & 0 & 0 & 10 \end{array}\right]$$

(5) $$K_{i}=P_{i|i-1}H^{T}S_{i}^{-1}$$

(6) $$P_{i|i}=(I-K_{i}H)P_{i|i-1}{\left(I-K_{i}H\right)}^{T}+K_{i}RK_{i}^{T}$$

(7) $$y^{i}=d^{t}-H{\hat{t}}_{paired}^{i|i-1}$$

(8) $${\hat{t}}_{paired}^{i|i}={\hat{t}}_{paired}^{i|i-1}+K_{i}y^{i}$$

where *S*_*i*_ is the covariance matrix of the measurement residuals for the *i*th frame and *H* is the measurement matrix that maps the tracker state variables to the measurement state variables (detection frame). *R* is the measurement error covariance matrix. *K_i_* is the Kalman filter gain in the *i*th frame, and *I* is the identity matrix. *y^i^* is the measured residual between the tracker's *a priori* estimated state of the *i*th image and the matched detection frame, and ${\hat{t}}_{paired}^{i|i-1}$ is the amount of the tracker's a posteriori estimated state.

#### Association and matching between frames

**Figure 4:**
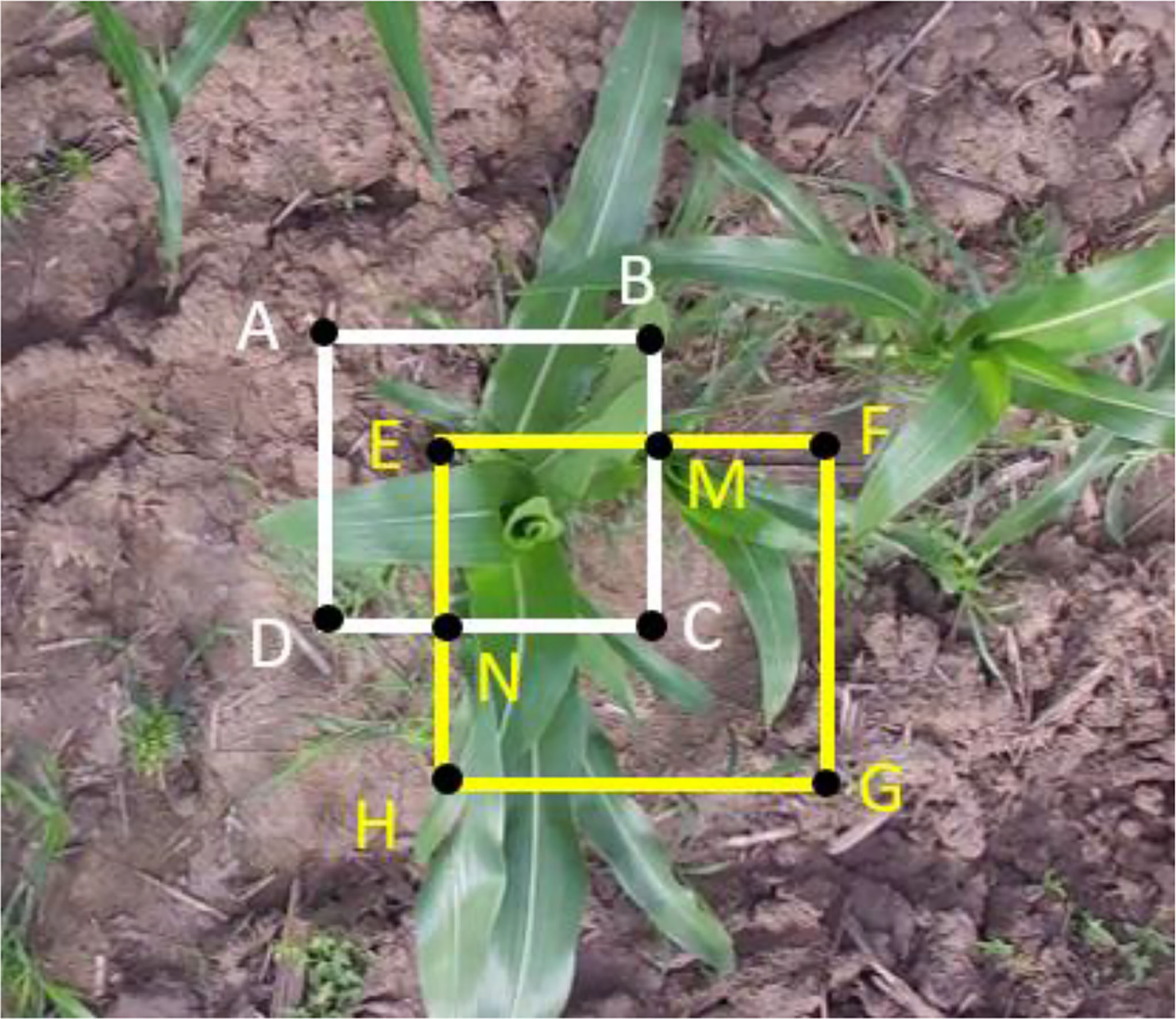
The schematic diagram of a maize plant detection and a tracking bounding box.

In the update process, the trackers in the (*i-1*)th frame and the detection results (***D_i_***) of the *i*th frame were used. Since the detection results could be valued as the ground truth for the current frame, it is necessary to match the detection results with the trackers and thus update the Kalman filter. In this study, the IoU-based Hungarian algorithm (Kuhn, 2005) was used to establish the association between the detection results and the trackers. **Figure 4** is a schematic diagram of a maize plant detection and a tracking bounding box. As shown in the figure, the white rectangle ABCD represents a maize plant bounding box predicted by the detector, and the yellow rectangle EFNM represents a maize plant bounding box predicted by the tracker. The overlap degree of the tracked bounding box and the detected bounding box is represented by formula (9). The closer the value of IoU is to 1, the higher the overlap and correlation between the detection bounding box and the tracking bounding box.


(9) $$IoU=\frac{S_{EMCN}}{S_{ABCD}+S_{EFGH}-S_{EMCN}}$$

Then the IOU was used in the Hungarian algorithm to calculate the cost matrix to establish the corresponding matching relationship between the maize plant tracked bounding box predicted by Kalman filter and the detection bounding box predicted by detectors. Assuming that maize plants have been tracked in the *i*th frame image denoted as *T*_*i*_={*T*_1_,*T*_2_,…,*T*_*m*_} , and maize plants detected in the (*i+1*)th frame image denoted as *D*_*i*_={*D*_1_,*D*_2_,…,*D*_*n*_} . The matching correlation matrix ***C*** is obtained by calculating the IoU of the tracking frame ***T*** and the detection frame ***D***. The calculation formula is shown in equation (10).


(10) $$\text{C}={\left(c_{i,j}\right)}_{mxn}=IoU(T,D)$$

The threshold *T*_*thresh*_ was set to process the matching incidence matrix ***C*** to obtain the result matrix ***R***. The processing formula is shown in equation (11).


(11) $$R={\left(r_{i,j}\right)}_{mxn}=\{\begin{array}{c} 0,\;c_{i,j}<T\\ 1,\;c_{i,j}>T \end{array}$$

In the formula, *T_thresh_* is equal to 0.3. when *r_i,j_* is 1, it means that the *i*th tracked maize plant is successfully associated with the *j*th detected maize plant. At the same time, it should be ensured that each tracked maize plant can only be associated with one detected maize plant. That is, equation (12) needs to be met.


(12) $$max\sum_{i=1}^{M}\sum_{j=1}^{N}c_{i,j}r_{i,j}\;s.t.(\sum_{i=1}^{M}r_{i,j}=1,\sum_{j=1}^{N}r_{i,j}=1)$$

#### Trackers update

After the matching of detection bounding boxes and trackers, detection bounding boxes (***D_i_***) and trackers (***T_i-1_***) can be divided into three categories: trackers associated with detection boxes, unmatched trackers, and unmatched detection bounding boxes. The trackers associated with detection boxes will be used in the update process. As for unmatched detection boxes, a new tracker will be created for each of them separately and will be added to the existing collection of trackers. For every unmatched tracker, its ***V_lost_*** will be increased by 1, which means it loses the target once. When the cumulative number of lost targets reaches the set threshold ***T_lost_***, it will be removed from the tracker set.

Since one tracker theoretically corresponds to one maize plant, the number of trackers is the number of maize plants. However, because the detection model may miss or misdetect, this will cause errors in the number of trackers and eventually lead to errors in the count of maize plants. For the missed detection problem of the detector, this study solves this problem by adding a parameter threshold ***T_lost_*** to the algorithm. When the missing detection of the detector causes the unmatched tracker appears, the ***V_lost_*** of the tracker will be increased by 1, which means that the tracker loses the target once. When the ***V_lost_*** reaches the set threshold ***T_lost_***, it will be removed from the tracker set. For the problem of false detection problem, the algorithm judges by setting the threshold ***T_life_***. Only when the cumulative number of tracker existences of a plant is greater than the threshold ***T_life_***, it will be regarded as a valid count.

### A quantitative statistical method based on cross-line counting

**Figure 5:**
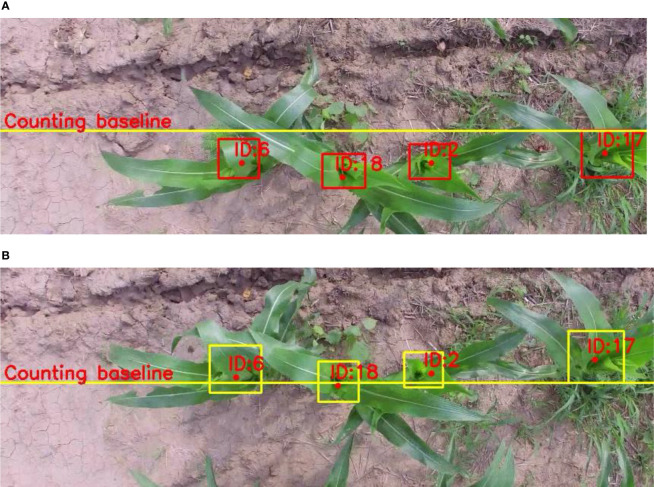
Demonstration of the counting baseline for counting. The yellow line is the counting baseline. The top shows the *n* th frame; the bottom shows the *n+i* th frame. **(A, B)** shows the n-th frame and n+1 th frame, respectively.

If the detection model misses a maize plant in several frames and then detects it again in another frame, the original tracking ID will be discarded and then a new ID will be created. When maize plants appear at the edge of the image, the view of the center of plants is prone to distortion. At this time, the performance of the tracker and detector would be affected by this. Therefore, a counting baseline was defined in the image to improve the counting accuracy. As shown in **Figure 5**, the counting baseline (the yellow line) is defined at the center (1/2 height) of the image. The counting baseline served as a reference line to count maize plants. The tracked bounding box would be regarded as a valid count when it crosses the counting baseline (in **Figure 5B**). At the same time, the color of the tracking box will change from red to yellow, indicating that the tracker has been counted.

## Test results and discussion

### Model training and testing

The python version and framework used were Python 3.8 and Pytorch 1.5.0, respectively. Ubuntu 16.04 was used with the Intel Core I7 6700K processor (64GB RAM) and the Nvidia GeForce RTX 3090. CUDA 10.1 parallel computing framework and CUDNN 7.6 deep neural network acceleration library were used. The batch size and epochs were set to 24 and 300, respectively. Other hyperparameters used the default values given by the official website. A pretrained weight trained on Microsoft Common Objects in Context (MS COCO) dataset (Lin et al., 2015) was used to initialize the weight of the model. In order to validate the performance of the algorithm, precision, recall rate, missed rate, and average precision (AP) are used to evaluate the trained model. The calculation formulas are as follows:


(13) $$P=\frac{TP}{TP+FP}$$

(14) $$R=\frac{TP}{TP+FN}$$

(15) $$M=\frac{FN}{TP+FN}$$

(16) $$AP=\int_{0}^{1}P(R)dR$$

where *P* is the precision, *R* is the recall, *M* is the miss detection rate, *TP* is the number of maize correctly detected by the model, *FP* is the number of backgrounds misclassified as maize plants and *FN* is the number of maize misclassified as background. Since the category detected in this study is only the maize plant, the AP (average precision) is equivalent to the commonly used mAP (mean average precision).

### Detection results of the model on maize plants

The trained Faster RCNN, SSD, YOLOV5, and SE-YOLOV5 models were tested on the test dataset respectively. The results are shown in **Table 2**. Comparing in terms of accuracy and speed in **Table 2**, it can be seen that the YOLOV5 series models are superior to the SSD model in both accuracy and speed. Although the YOLOV5 series models are comparable to Faster RCNN in terms of accuracy, their speed is more than 7 times that of Faster RCNN. The mAP of SE-YOLOv5m is 1.21 higher than that of YOLOv5m. Meanwhile, the model size and the average detection speed of the SE-YOLOv5m model are close to that of the YOLOv5m model. Thus, the SE-YOLOv5m network model was adopted in this experiment after considering the detection accuracy and the lightweight requirement of the network.

**Table 2:** Comparison of different detection models on the test set.

Models	mAP (%)	Average detection speed (ms)	Model size (MB)
YOLOV5m	90.24	20.3	40.8
SE-YOLOV5m	91.45	20.4	42.7
SSD	78.32	44.2	82.78
Faster R-CNN	91.88	180.4	110.8

### Accuracy evaluation of the model under different weed proportions

**Figure 6:**
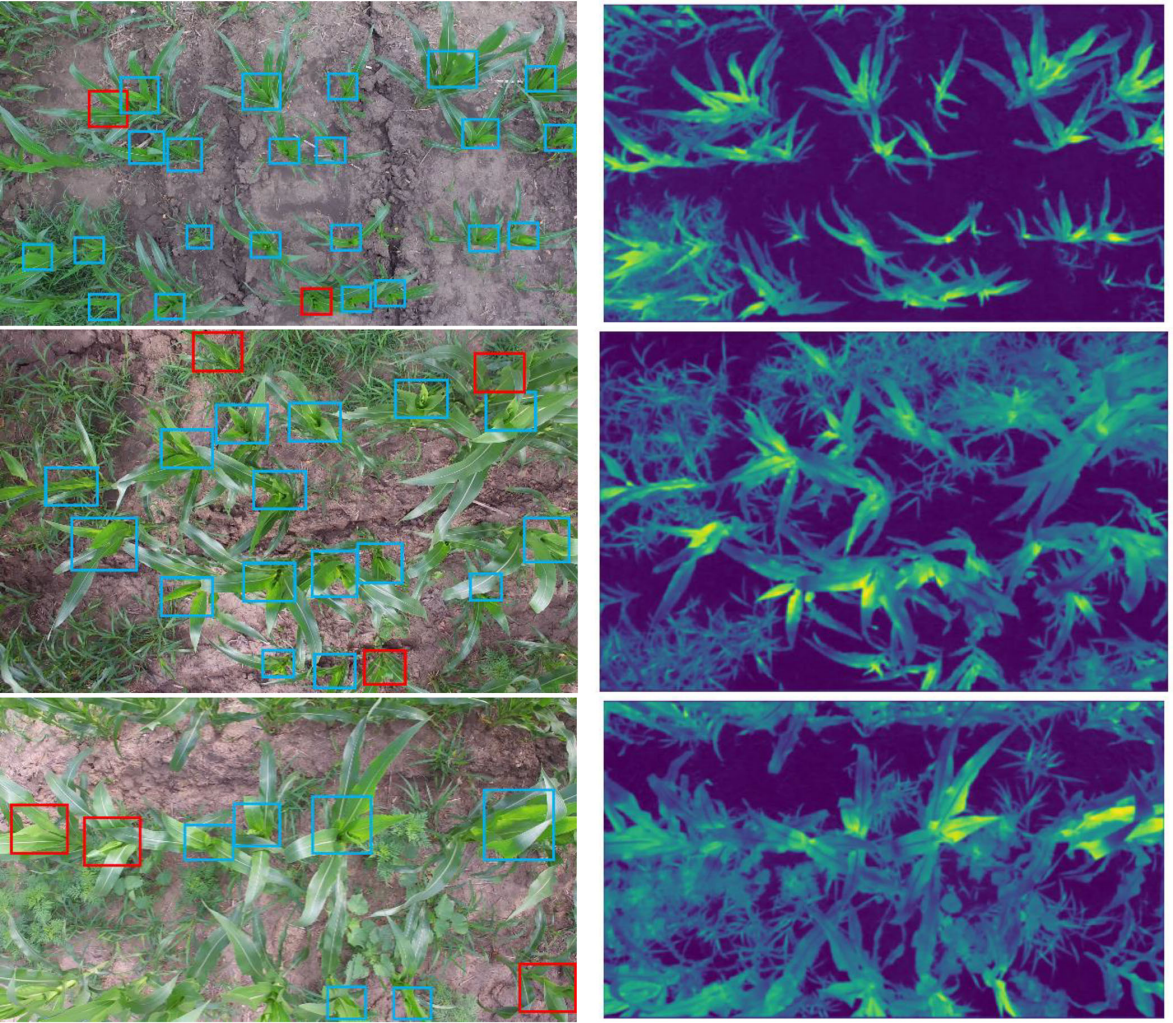
Detection results and feature maps of SE-YOLOV5m under different weed proportions. The left column shows detection results; the right column shows the corresponding feature maps of the last layer in the first C3_2 module of SE-YOLOV5m. From top to bottom are representative images with weed proportions less than 30%, between 30% and 60% and more than 60%, respectively. In the figure, the blue boxes and red boxes are the *TP* and *FN*.

Because weeds are easy to grow in the seedling stage of maize, excessive weeds may even affect the growth of maize. Therefore, the complex environment in this study mainly refers to different weed proportion. The presence of weeds in some areas of the maize field may have an impact on the accuracy of the detection model. Therefore, the above test set was split into three parts according to different the proportion of weeds in the field: a dataset with a weed proportion less than 30% (denoted by A), a dataset with a weed proportion between 30% and 60% (denoted by B) and a dataset with the weed proportion greater than 60%. Among them, the number of pictures in test sets A, B, and C are 80, 90, and 50, respectively. The tested models are the above-mentioned SE-YOLOV5 model and other state-of-art models. The detection results are shown in **Table 3**. The test sample results under different weed proportions are shown in **Figure 6**. It can be seen from the table and the figure that different weed proportions in the field have no significant influence on the detection accuracy of the maize plant detection models. The reason may be that weeds are different from maize plants in color, texture, and shape, so detection models are able to distinguish weeds from maize plants more directly. Then, the convolution feature maps of maize plants are visualized in **Figure 6** to further analyze the reasons. It can be seen in **Figure 6** that the features extracted by the model can well distinguish weeds from maize plants. In addition, it can also be seen from the FN samples in the figure (red boxes in the first column) that when the core leaves of the maize plant are partially obscured or the view of the central leaves is skewed, the model would have a certain degree of missed detection. It can also be seen from the corresponding feature map that the model can not extract effective feature information to distinguish maize plants at this time.

**Table 3:** Comparison of detection results under different weed rates.

Models	Dataset A	Dataset B	Dataset C
YOLOV5m	91.24	91.26	90.46
SE-YOLOV5m	92.68	92.65	92.02
SSD	79.24	79.12	78.62
Faster R-CNN	92.88	92.88	92.88

### Counting accuracy regression analysis and evaluation

**Figure 7:**
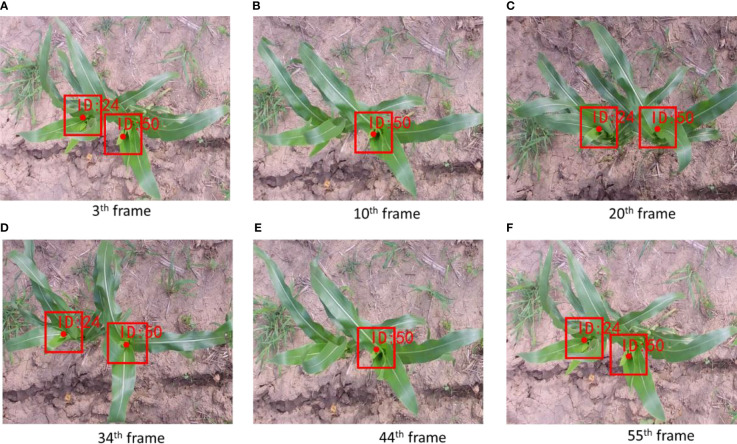
Tracking example of intermittently detected maize plant. **(A–F)** shows the result in the 3th frame, 10th frame, 20th frame, 34th frame, 44th frame and 55th frame, respectively.

Videos in the counting dataset were segregated into 23 video clips for evaluating the developed counting algorithm, and they were individually counted by three researchers. Each video clip represented an approximately 3 m long segment in the videos. Frame rate and length of each video were about 30 frames per seconds (FPS) and 10 s, respectively. Then, the counting results were averaged to obtain the actual number of maize plants in the corresponding video. The counting algorithm based on SE-YOLOV5m was tested on the videos. Based on the proposed algorithm, the corn plant video tracking experiment was carried out. **Figure 7** is an example of tracking a maize plant video sequence based on the proposed algorithm. As can be seen in the figure, the No. 24 corn plant has been detected and tracked for 55 consecutive frames in the video. Due to the disturbance of wind, the key features of the No. 50 maize plant are occluded in the 10^th^ and 44^th^ frames, which leads to intermittent missed detection. The algorithm can still track the target in subsequent images and keep the original ID unchanged, which is because ***T_lost_*** is set in this study. When ***T_lost_*** is not set, the algorithm cannot track the target in subsequent images. Therefore, it can be seen that although there is a short-term missed detection phenomenon in the video, the algorithm in this study could still effectively track maize plants.

**Figure 8:**
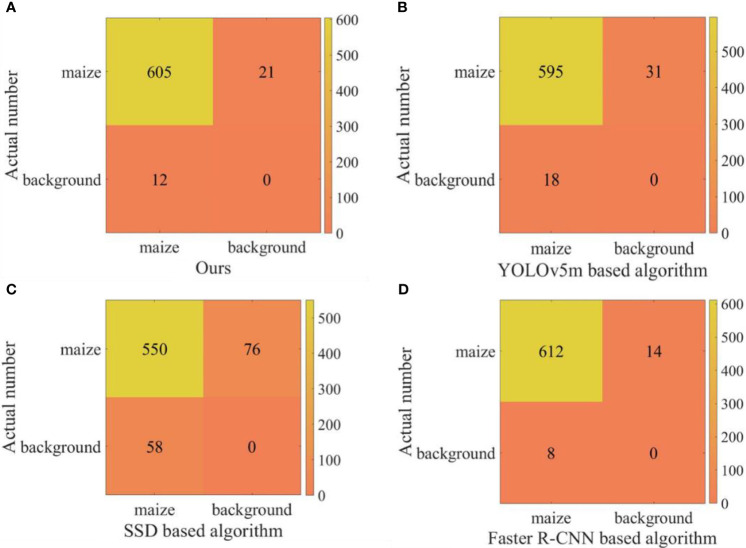
The confusion matrix of of the four algorithms. **(A–D)** shows the confusion matrix of Ours algorithm, YOLOv5m based algorithm, SSD based algorithm and Faster R-CNN based algorithm, respectively.

**Figure 9:**
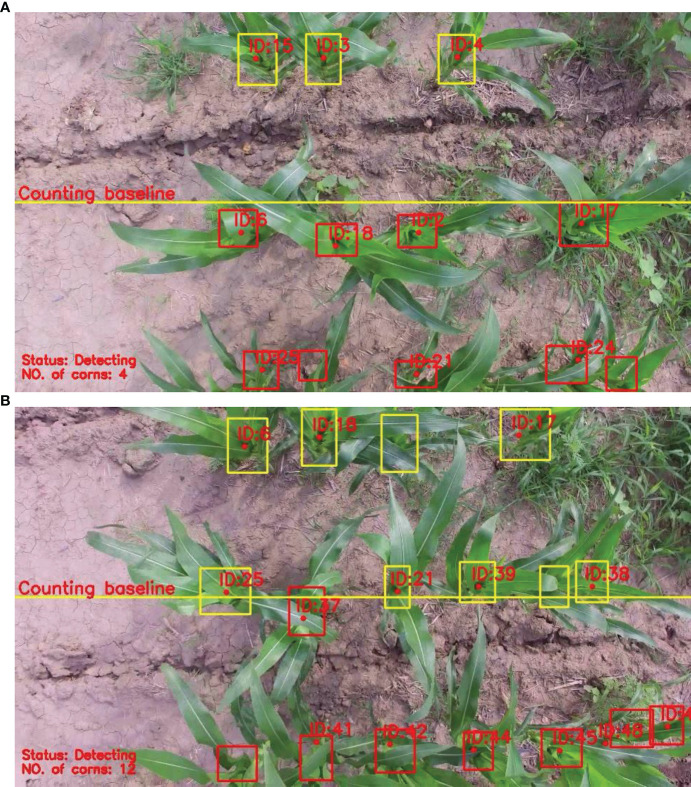
Visual video counting result of the algorithmic. The number on the bounding box is the ID of the mazie plant being tracked, and the solid point is the center of the mazie plant being tracked. The test video contains a total of 311 frames. In the figure, the 104 and 241 are typical frames. **(A, B)** shows the result in the 104th frame and 241th frame, respectively. Corrections has been post in the Production Forum.

In order to verify the performance of the proposed algorithm, 23 videos in the counting dataset are used as experimental data for comparative experiments. The comparison models were to replace the SE-YOLOv5m model in the proposed algorithm with the trained YOLOv5m, SSD, and Faster R-CNN models, respectively. The confusion matrix was used as the evaluation index to compare the performance of the four algorithms quantitatively. The experimental results are shown in **Figure 8**. At the same time, the frame rates of the proposed algorithm and the algorithm based on the above three models are 28.2, 28.4, 20.2, and 5.2, respectively. It can be seen from the results that the running speed of the proposed algorithm is similar to that of the counting algorithm based on YOLOv5m, but its accuracy is higher. The performance of the counting algorithm based on SSD is poor, mainly because the SSD model has low detection accuracy, which can also be confirmed in **Table 2**. Compared with the counting algorithm based on Faster R-CNN, the proposed method is faster on the basis of comparable accuracy. Although the counting algorithm based on Faster R-CNN performs well in terms of accuracy, there is still a lot of room for optimization in terms of running speed. Therefore, according to the comprehensive analysis of accuracy and speed, we can see that the performance of the proposed algorithm is the best among the four methods. Taking one of the videos as an example, there are a total of 311 frames of a video collected by UAV, and the statistical results are shown in **Figure 9**. Among them, the statistical result of the number of the 104th frame is 4, and the statistical result of the number of the first 241 frames is 14. During the process of tracking and matching, the number of some maize plants was lost at the edge (some plants don’t have ID numbers), but the cross-line counting method effectively solved this problem. It shows that under the interference of ground weeds and wind, the algorithm in this study could accurately count the number of maize plants.

## Conclusion

(1) The YOLOV5m model which incorporates a channel attention mechanism (SENet) was constructed to achieve effective detection of maize plants in a complex background. The mAP of the SE-YOLOV5m model on the test set was 90.66% (IoU 0.5), indicating the effectiveness of the SE-YOLOV5m model for detecting maize plants. The proposed SE-YOLOV5m model was able to infer at 20.4 ms on a GPU on an image with the size of 960 pixels × 540 pixels, which have the potential to be applied to embedded terminals. Evaluation under different weed proportions shows that different weed proportions in the field have no significant influence on the detection accuracy of the maize plant detection models.

(2) A deep-learning-based method for counting maize plants in a field was proposed, which used an improved YOLOV5 model with a Kalman filter. The mazie plant counting method proposed in this paper was compared with the counting algorithms based on YOLOv5, SSD and Faster R-CNN algorithms. The test results show that the proposed method is significantly better than the SSD-based algorithm in terms of accuracy and speed. Its speed is similar to that of the counting algorithm based on YOLOv5, but its accuracy is higher. Its accuracy is similar to that of the algorithm based on Faster R-CNN, but the frame rate is about 23 higher. Therefore, the proposed counting method is an effective method to achieve fast and accurate counting of the number of maize plants. In addition, the detection methods and annotated images used in this study could be used by the other researchers and engineers to further develop maize plants detection and counting methods.

## Data availability statement

The raw data supporting the conclusions of this article will be made available by the authors, without undue reservation.

## Author contributions

YL: conceptualization, methodology, writing—original draft. ZB: resources, software, data curation, and investigation. JQ: methodology, supervision, writing—review and editing, and funding acquisition. All authors contributed to manuscript revision, read, and approved the submitted version.

## Funding

This study was supported by the National Natural Science Foundation of China (31971783) and the Central Public-Interest Scientific Institution Basal Research Fund (1610212022004).

## Conflict of interest

The authors declare that the research was conducted in the absence of any commercial or financial relationships that could be construed as a potential conflict of interest.

## Publisher’s note

All claims expressed in this article are solely those of the authors and do not necessarily represent those of their affiliated organizations, or those of the publisher, the editors and the reviewers. Any product that may be evaluated in this article, or claim that may be made by its manufacturer, is not guaranteed or endorsed by the publisher.
